# Improving outcomes in atherosclerotic renovascular disease: importance of clinical presentation and multi-disciplinary review

**DOI:** 10.1007/s40620-024-01902-1

**Published:** 2024-04-09

**Authors:** Áine M. de Bhailis, Edward Lake, Constantina Chrysochou, Darren Green, Rajkumar Chinnadurai, Philip A. Kalra

**Affiliations:** 1 Department of Renal Medicine, Salford Royal NHS Foundation Trust, Salford, M6 8HD UK; 2 Faculty of Biology, Medicine and Health, University of Manchester, Manchester, UK; 3 Department of Vascular Interventional Radiology, Manchester University NHS Foundation Trust, Manchester, UK

**Keywords:** Hypertension, Renovascular disease, Renal artery stenosis, Revascularization

## Abstract

**Background and objectives:**

Atherosclerotic renal artery stenosis may cause hypertension, chronic kidney disease and heart failure, but large randomized control trials to date have shown no major additional benefit of renal revascularization over optimal medical management. However, these trials did not consider outcomes specifically in relation to clinical presentations. Given that atherosclerotic renal artery stenosis is a heterogenous condition, measures of success likely differ according to the clinical presentation. Our retrospective study objectives were to determine the effects of revascularization when applied to specific clinical presentations and after careful multi-disciplinary team review.

**Methods:**

All patients presenting to our centre and its referring hospitals with radiological findings of at least one renal artery stenosis > 50% between January 2015 and January 2020 were reviewed at the renovascular multi-disciplinary team meeting with revascularization considered in accordance with international guidelines, notably for patients with anatomically significant renal artery stenosis, adequately sized kidney and presentations with any of; deteriorating kidney function, heart failure syndrome, or uncontrollable hypertension. Optimal medical management was recommended for all patients which included lipid lowering agents, anti-platelets and anti-hypertensives targeting blood pressure ≤ 130/80 mmHg. The effect of revascularization was assessed according to the clinical presentation; blood pressure and number of agents in those with renovascular hypertension, delta glomerular filtration rate in those with ischaemic nephropathy and heart failure re-admissions in those with heart failure syndromes.

**Results:**

During this 5-year period, 127 patients with stenosis ≥ 50% were considered by the multidisciplinary team, with 57 undergoing revascularization (17 primarily for severe hypertension, 25 deteriorating kidney function, 6 heart failure syndrome and 9 for very severe anatomical stenosis). Seventy-nine percent of all revascularized patients had a positive outcome specific to their clinical presentation, with 82% of those with severe hypertension improving blood pressure control, 72% with progressive ischaemic nephropathy having attenuated GFR decline, and no further heart failure admissions in those with heart failure. Seventy-eight percent of patients revascularized for high grade stenosis alone had better blood pressure control with 55% also manifesting renal functional benefits.

**Conclusions:**

Multi-disciplinary team discussion successfully identified a group of patients more likely to benefit from revascularization based on 3 key factors: clinical presentation, severity of the renal artery lesion and the state of the kidney beyond the stenotic lesion. In this way, a large proportion of patients can clinically improve after revascularization if their outcomes are considered according to the nature of their clinical presentation.

**Graphical Abstract:**

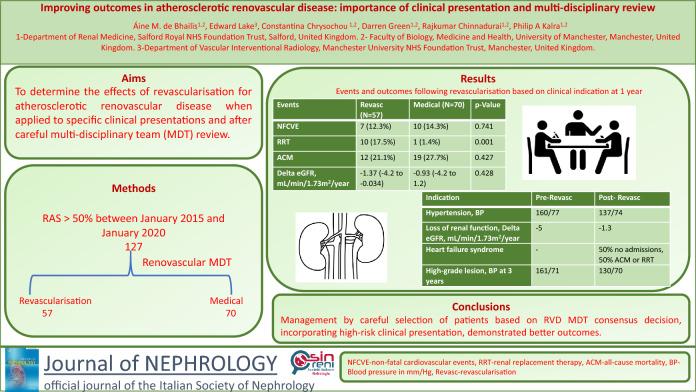

## Introduction

Atherosclerosis is the leading cause of renal artery stenosis. The epidemiology of atherosclerotic renovascular disease varies depending on the cohort studied and the screening tool used. The prevalence amongst the overall US Medicare population aged over 67 years was 0.54% with an annual incidence of 0.37% for new cases [1] However, the prevalence increases in the presence of atherosclerotic disease in other vascular beds, being 10.5% in those with coronary artery disease, 25.5% in those with peripheral vascular disease and up to 54.1% in those with congestive heart failure [2].

Atherosclerotic renovascular disease can lead to three main clinical presentations which often co-present: renovascular hypertension, ischaemic nephropathy (with renal functional impairment), and cardiac destabilisation syndromes. Large, randomised control trials (RCTs) have to date failed to show an additional benefit of renal revascularization to medical management in those with atherosclerotic renovascular disease [3–5]. However high-risk patients were underrepresented in these RCTs, and other non-randomised studies have illustrated clinical benefits in those with high-risk clinical presentations such as rapidly declining renal function, severe hypertension and flash pulmonary oedema [6].

A multi-disciplinary team approach seems appropriate to select patients for revascularization based upon consideration of the nuances of their clinical presentation, the severity of the stenotic lesion and prior kidney damage as indicated by level of proteinuria or renal size. Such an approach has been illustrated by the group in Lyon, France in which Sens et al. examined outcomes for 49 atherosclerotic renovascular disease patients diagnosed over a 2-year period from April 2013 to April 2015, who were discussed by the multidisciplinary team [7] The multidisciplinary team decision for either medical treatment or revascularization was compared to international guidelines, and blood pressure control and renal function at one year was compared to baseline. Of the 23 patients selected for renal stenting, 83% had controlled blood pressure at 12 months compared to 42% receiving medical therapy alone, and they also required a reduced number of antihypertensive agents ( – 1.00 ± 1.03, *p* = 0.001) at follow up.

For over three decades we have carefully examined the outcomes of patients with atherosclerotic renovascular disease undergoing revascularization in our centre [8–10]. Acknowledging the results from the RCT, that revascularization is inappropriate for the majority of asymptomatic patients with atherosclerotic renal artery stenosis, but also the observational data that certain patients do definitely gain benefit [6], we have been conducting a monthly multidisciplinary team review of all cases with atherosclerotic renovascular disease and higher-grade renal artery stenosis presenting to our centre since 2015. In this observational cohort study, we describe the clinical decision-making process in deciding suitability for revascularization and emphasise the importance of evaluating the most appropriate outcome measure that relates to an individual patient’s clinical presentation.

## Material and methods

### Study design and population

All patients presenting to our centre and its referring hospitals with radiological findings of at least one renal artery stenosis lesion > 50% between January 2015 and January 2020 were reviewed at the renovascular multidisciplinary team meeting in accordance with international guidelines from both the European Society of Cardiology (ESC) [11] and American Heart Association (AHA) [12].

The diagnosis of atherosclerotic renal artery stenosis was established based on computed tomography angiographic or magnetic resonance angiographic findings consistent with a renal artery lesion of ≥ 50% of the lumen based on visual assessment. Computed tomography or contrast enhanced magnetic resonance angiography were the preferred screening investigations, however, in those with stage 4 chronic kidney disease (CKD) or worse (i.e. glomerular filtration rate (GFR) < 30 ml/min), non-contrast magnetic resonance angiography was utilised. The sequence used for non contrast magnetic resonance angiography was a 3D steady state free precession with in-flow inversion recovery (Inhance, GE Healthcare).

Patients with findings consistent with fibromuscular dysplasia or large vessel vasculitis were excluded in this analysis. Patients with a significant atherosclerotic lesion and a clinical phenotype of severe hypertension, decompensated heart failure syndrome or deteriorating renal function were referred to the nephrology team by specialists in cardiology, nephrology, stroke medicine and general medicine and then discussed at the multidisciplinary team meeting along with the relevant imaging.

### Description of the multi-disciplinary meeting

The multidisciplinary team meeting was held once a month. The team consisted of at least two nephrologists, an interventional radiologist and, when required, a referral was made to vascular surgery. Many of the referrals originated from colleagues in cardiology reviewing patients with hypertension or cardiomyopathy.

The discussion of each patient’s case involved a review of their electronic patient record taking note of the clinical presentation, their medical history and co-morbidities, blood pressure, laboratory data (notably prior renal function changes and level of proteinuria), and any cardiac history/complaints.

The interventional radiologist presented the radiological findings and description of the renal artery stenosis lesion and whether or not it would be amenable to renal artery stenting. The referring physician highlighted whether the patient was asymptomatic or whether a clinical indication for revascularization existed. The decision regarding whether to proceed to revascularization was based on three components, all of which were considered relevant:

- High-grade renal artery stenosis lesion.
- the state of the kidney beyond the renal artery stenosis (kidney size ≥ 8 cm, and review of level of proteinuria).
- the clinical presentation of the patient.

Clinical aspects favouring revascularization included resistant hypertension (blood pressure ≥ 140/90 despite the use of three or more anti-hypertensive agents), deteriorating renal function not otherwise explained, sudden onset pulmonary oedema or recurrent admissions for heart failure, a renal artery stenosis lesion ≥ 75% affecting a solitary kidney. A high-grade renal artery stenosis was a lesion > 75% of the lumen diameter or those with radiologic evidence suggesting compromised blood flow [13].

Patients were revascularized according to priority, with inpatients with acute kidney injury (AKI) or those with severe renal artery stenosis and worse clinical picture being expedited.

Proteinuria is a significant marker of established renal parenchymal damage and has been shown as an independent predictor of worse outcomes in those with ischaemic nephropathy. Significant albuminuria (urine albumin creatinine ratio, (ACR), > 30 mg/mmol) or proteinuria (urine protein creatinine ratio, (PCR), > 50 mg/mol) are independent predictors of worse outcomes in patients revascularized for hypertension [14].

Clinical aspects that would argue against revascularization were clinical frailty, multiple co-morbidities, lesions of 100% without clinical features of renal artery sclerosis or an atrophic kidney with length < 8 cm.

Multiple comorbidity refers to disease in several organ systems, which in the opinion of the managing physician implied that a revascularization procedure would be high risk or futile. Frailty was assessed by the Clinical Frailty Scale [15].

Each member of the multi-disciplinary team gave their opinion regarding the risks and potential benefits of revascularization followed by a comprehensive discussion to reach a consensus. If the computed tomography or magnetic resonance angiography image quality was inadequate, further imaging was arranged with a planned discussion at a subsequent meeting.

If the decision was to proceed with revascularization, then the patient was notified, and the interventional radiologist made arrangements to meet with them to counsel on risks and benefits of the procedure before proceeding with renal artery angioplasty and stenting at a later date. Those patients with no clinical indication for revascularization were informed of the decision and if they had significant anatomical renal artery stenosis, they remained under long term follow up in our centre’s renovascular clinics, where note would be taken of any change in clinical circumstances that might lead to a change of plan.

### Baseline and follow-up data

For this study, all medical records and medical correspondence of the patients considered in the renovascular multidisciplinary team meeting were reviewed. Resistant hypertension was defined as blood pressure > 140/90 mmHg despite three different classes of anti-hypertensive agents, one of which was a diuretic.

Renal function was based on estimated glomerular filtration rate (eGFR) using the Chronic Kidney Disease-Epidemiology Collaboration formula (CKD-EPI). Deteriorating renal function was defined as a decrease in eGFR of ≥ 30% from baseline in the absence of an alternative aetiology.

Blood pressure readings were the most recent clinical measurement at the time of multidisciplinary team discussion. Our standard protocol is to obtain three consecutive blood pressure readings when sitting quietly for a period of ten minutes with the lowest reading being recorded. Follow-up data included blood pressure readings taken at our unit 1 month post-revascularisation and annually thereafter. In the non-revascularized group, annual blood pressures were recorded from our centre’s electronic patient record or clinical correspondence from referring units. For patients with bilateral renal artery stenosis, the worst affected side was recorded.

For each patient, the decision of either medical management or revascularization made during the multidisciplinary meeting was noted and compared to international guidelines.

Clinical events recorded at annual follow-up included cardiovascular events and renal events in the preceding 12 months. Non-fatal cardiovascular events included acute coronary syndrome (myocardial infarction/unstable angina), cerebrovascular event (stroke or transient ischaemic event), admission for heart failure syndrome or new-onset atrial fibrillation. Renal events included severe hypertension, acute kidney injury, requirement for renal replacement therapy (RRT) (temporary or long term), transplantation or nephrectomy.

### Treatment

Medical treatment of all patients was optimised according to current guidelines which included lipid-lowering agents, antiplatelet agents, anti-hypertensives agents targeting clinic blood pressure of ≤ 130/80 with the use of renin-angiotensin blocking agents where tolerated, and lifestyle modifications such as smoking cessation [16]. Patients were reviewed at regular intervals ranging from 3–6 months.

### Renal artery revascularization protocol

Elective renal artery stenting procedures were usually carried out on a day case basis. The standard renal artery stenting technique consisted of ultrasound-guided common femoral arterial access secured with a 45 cm 5F Flexor ® sheath (Cook Medical). Following a renal angiogram, the stenotic lesion was crossed with an 0.35 Glidewire ® (Terumo) and catheter. An 0.18 SV-5 (Cordis) support wire would be placed in the renal artery distally. The stenotic lesion was then primarily stented with a Palmaz blue ™ (Cordis) cobalt-chromium balloon mounted bare metal stent with 1:1 sizing with the normal renal artery. Variations include arterial access from the arm (brachial or radial artery); use of a steerable sheath (OSCOR) for challenging procedures, and pre-dilatation of the lesion with a small angioplasty balloon for very tight lesions. Note was taken of any immediate complications of revascularization at the time of the procedure (e.g. dissection of the renal artery, puncture site haematoma), or complications occurring in the days post-procedure (e.g. cholesterol embolization).

At yearly follow-up, the following data were collected: systolic and diastolic blood pressure, number of anti-hypertensive agents, eGFR, urine PCR, haemoglobin, the incidence of major cardiovascular events (myocardial infarction or coronary revascularization, stroke, heart failure admission or peripheral vascular disease procedure), renal events, and mortality.

### Statistical analysis

In the descriptive analysis continuous variables were reported as median and interquartile range after checking for normality of distribution with the Mann–Whitney *U* test. Categorical variables were expressed as number and percentage with Chi-square test used as required.

The annual rate of decline in eGFR was calculated by using all the available eGFRs between the study start and end date using linear regression analysis. Only patients with a minimum of three eGFR values and one year follow-up data were included in the linear regression analysis. The association between revascularization and all-cause mortality was shown in Kaplan–Meier plots.

Throughout the analysis a *p* < 0.05 was considered as statistically significant. All analyses were carried out by SPSS Version-24, registered with the University of Manchester.

### Ethical considerations

The study complies with the declaration of Helsinki and was registered with the Research and Innovation department of the Northern Care Alliance NHS Group (Reference Number: 22HIP25), who approved the methodological protocol as outlined above. As this was a retrospective observational study using measurements routinely collected and fully anonymized data, the need for individual patient consent was waived by the Research and Innovation review committee, who granted study approval.

## Results

### Patient characteristics

During the 5-year review period, 127 patients with radiologically-proven atherosclertotic renovascular disease (renal artery stenosis ≥ 50%) were reviewed in the multidisciplinary team meeting. A similar number of patients with insignificant anatomical renal artery stenosis were considered by the multidisciplinary team but only in relation to medical vascular protective management.

Overall, the most common clinical indication for screening for atherosclerotic renovascular disease was unexplained deterioration in renal function, or acute kidney injury in the setting of renin-angiotensin-aldosterone system inhibition, these two presentations accounting for 52% (62) of the patients. Hypertension accounted for 37%, with resistant hypertension accounting for 11% (14 patients). Cardiac destabilisation syndromes accounted for 11% of those screened.

A computed tomography or magnentic resonance renal angiogram was performed in 54 patients and 72 patients, respectively, with one patient undergoing formal catheter angiography. Of the 72 patients who underwent magnetic resonance angiography screening, 4 had contrast imaging and 68 had a non-contrast magnetic resonance angiography.

After review of each patient’s severity of renal artery stenosis, kidney size, clinical presentation and current clinical characteristics, 70 (55%) patients were assigned to optimal medical treatment alone and 57 patients (45%) underwent revascularization in conjunction with optimal medical treatment. All patients were treated according to the multidisciplinary team consensus decision.

Baseline characteristics are summarised in Table 1. The median age was 72 years, eGFR 42 ml/min/1.73m^2^, uPCR 24 mg/mmol and blood pressure 156/77 mmHg. There was no significant difference between the medical and revascularization groups except for age, history of hypertension and the presence of resistant hypertension as defined by treatment with more than three anti-hypertensive agents.

**Table 1:** Comparison of baseline characteristics between the patients managed with revascularization and medical management as discussed at the multidisciplinary team meeting

Variable	Total *N* = 127	Revascularization *N* = 57	Medical *N* = 70	*p*-value
Age, years	72 (65–77)	68 (61–76)	73 (66–79)	**0.019**
Gender, female	76 (59.8%)	35 (61.4%)	41 (58.6%)	0.746
BMI, Kg/m^2^	27.8 (24.6–31.5)	27.1 (22.6–31.8)	28.2 (25.4–32.8)	0.290
Systolic BP, mm Hg	156 (136–178)	160 (139–181)	153 (134.5–174)	0.328
Diastolic BP, mm Hg	77 (68–89)	77 (66.5–99.5)	77 (70–87)	0.778
Smoking history	82 (64.6%)	33 (57.9%)	49 (70.0%)	0.156
Hypertension	120 (94.5%)	57 (100%)	63 (90%)	0.014
DM	40 (31.5%)	13 (22.8%)	26 (37.1%)	0.129
IHD	58 (45.7%)	25 (43.9%)	33 (43.7%)	0.712
HF	47 (37%)	21 (36.8%)	26 (37.1%)	0.972
CVA	25 (19.7%)	13 (22.8%)	12 (17.1%)	0.425
PAD	35 (27.6%)	17 (29.8%)	18 (25.7%)	0.606
Rt renal length, cm	8.7 (10–10.7)	10 (8.75–10.8)	9.9 (8.7–10.5)	0.920
Lt renal length, cm	9.9 (8.8–11)	9.6 (8.5–11.0)	10.0 (8.8–11.2)	0.680
RAS (% narrowing of lumen) –worst affected vessel	85 (70–95)	90 (70–95)	75 (70–95)	**0.026**
Presence of Bilateral disease	103 (81.1%)	45 (78.9%)	58 (82.9%)	**0.576**
≥ 3 anti-hypertensive agents	45 (35.4%)	29 (50.9%)	16 (22.9%)	**0.001**
RAASi	86 (67.7%)	42 (73.7%)	44 (62.9%)	0.194
Diuretic	76 (59.8%)	38 (66.7%)	38 (54.3%)	0.157
Cholesterol lowering agents	97 (76.4%)	46 (80.7%)	51 (72.9%)	0.301
Anti-platelet agents	58 (45.7%)	30 (52.6%)	28 (40.0%)	0.155
Warfarin	6 (4.7%)	3 (5.3%)	3 (4.3%)	0.796
DOAC	4 (3.1%)	1 (1.8%)	3 (4.3%)	0.417
Creatinine, micromol/L	126 (100–168)	127 (98–166)	125 (102–160)	0.605
eGFR, mL/min/1.73m^2^	42 (30–54)	40 (33.5–56)	43 (28.8–53.2)	0.443
UPCR, g/mol	24 (12–80)	36 (12.5–114)	21 (11–75)	0.440
Haemoglobin, g/L	125 (112–136)	123 (109–135)	125 (113–136)	0.240
Total cholesterol, mmol/L	4,3 (3.8–5.4)	4.4 (3.9–5.3)	4.3 (3.8–5.4)	0.907
Cholesterol: HDL ratio	3.4 (2.9–4.4)	3.5 (2.8–3.9)	3.4 (2.9–4.5)	0.691

Continuous variables are presented as median (inter quartile range) and p-Value by Mann–Whitney *U* Test. Categorical variables are presented as number (percentage) and *p*-value by Chi-square test

The bold values indicate a statistically significant *p*-value i.e. *p*-value of 0.05 or lower

*BMI* body mass index, *BP* blood pressure, *DM* diabetes mellitus, *IHD* ischaemic heart disease, *CCF* congestive cardiac failure, *CVA* cerebrovascular accident, *MDT* multidisciplinary team, *PVD* peripheral vascular disease. *RAASi* renin angiotensin aldosterone system inhibitors, *NOAC* novel oral anticoagulants, *uPCR* urine protein creatinine ratio. *HDL* high density lipoprotein, *LDL* low density lipoprotein

Missing BMI in 15 patients, Missing uPCR in 26 patients, Missing cholesterol in 62 patients, Missing cholesterol: HDL in 64 patients, Missing Hb in 9 patients, Missing renal length in 25 patients

### Adverse events in relation to revascularization procedures

Out of 57 patients who proceeded to have renal angioplasty and stenting, all but one had successful restoration of renal artery patency with the procedure. This patient underwent a bilateral revascularization attempt which was successful for one side but the contralateral revascularization was sub-optimal.

There were complications noted in a number of patients. Four (7%) patients had immediate complications: 2 had peri-renal haematomas secondary to renal artery injuries (3.5%) and two access site complications. Both immediate access site complications were of the brachial artery which had been used due to severely diseased ilio-femoral access vessels. Three (5.2%) patients had post-procedure cholesterol athero-embolization which stabilised. There were no contrast-induced AKI episodes recorded in our cohort. The complications are detailed in Table 2, but no patients suffered long-term harm.

**Table 2:** Complications incurred in the revascularization group

Complication	Number	Outcome	Hospital admission extended	Long term harm	Indication for revascularization
*Renal artery damage*	2				
Rupture with peri-renal haematoma		Conservative management	Yes	No	Hypertension
Left retro-peritoneal haematoma		Blood transfusion required	Yes	No	Heart failure
*Puncture site*	2				
Brachial pseudo-aneurysm		Conservative	No	No	Declining renal function
Puncture wound of brachial artery		Conservative	Yes	No	Heart failure
*Cholesterol embolization*	3				
Cholesterol embolization to lower limbs		Mottling of skin	No	No	Hypertension
Embolization to lower pole minor vessel		Monitored	No	No	Declining renal function
AKI secondary to embolization		Monitored as outpatient	No	No	Hypertension
*Other*	1				
Post procedural deep vein thrombosis		Anti-coagulated for 6 months	No	No	Hypertension

### Overall clinical outcomes of revascularized and medically treated patients

**Fig. 1:**
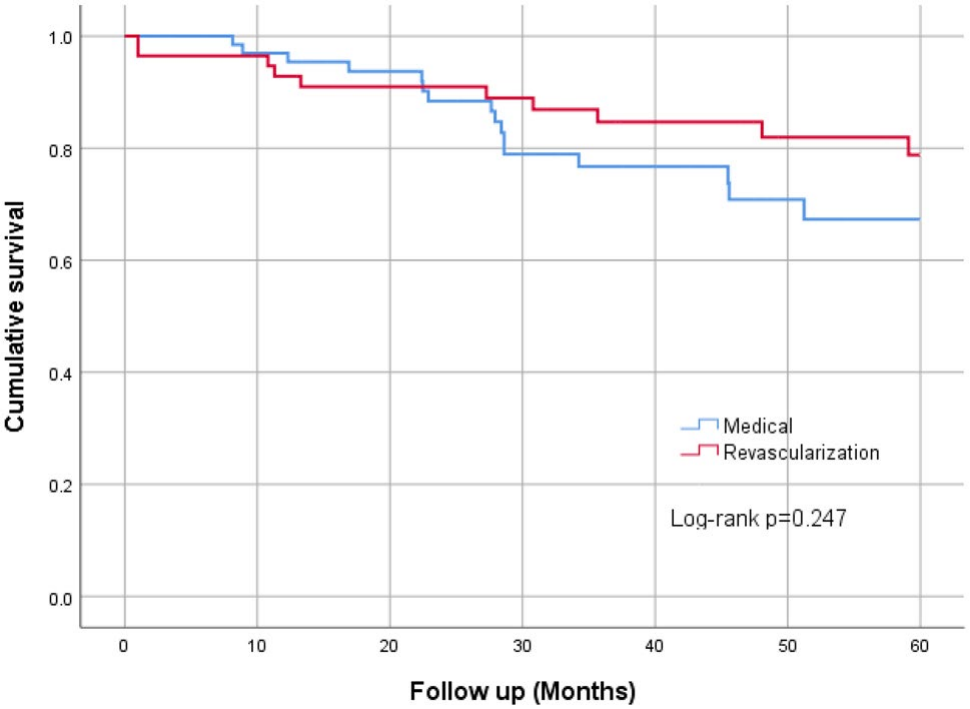
Kaplan Meier chart on all-cause mortality between the groups

When reviewing outcomes in the patients grouped according to treatment assignment without focusing on the reason for revascularization, during a median follow up of 42 (23–66) months there was no significant difference in the rate of non-fatal cardiovascular events between the two groups, with non-fatal cardiovascular events occurring at an overall rate of 3.4% per year (Table 3). Although average annual rates of loss of eGFR were similar (1.37 ml/min/year for revascularized patients compared to 0.93 ml/min/year) during follow up, 17.5% of patients in the revascularization group progressed to end-stage kidney disease (ESKD) requiring renal replacement therapy compared to only 1.4% of medically treated patients (*p* < 0.001). The explanation for this difference was thought likely due to pre-procedure slope of eGFR decline (see below). Mortality was similar in the two groups, with an annual mortality rate of 5% per year in revascularized patients compared to 9% per year in medically managed patients (Fig. 1).

**Table 3:** Comparison of outcomes between the groups

Event	Total *N* = 127	Revascularization *N* = 57	Medical Management *N* = 70	*p*-value
NFCVE	17 (13.4%)	7 (12.3%)	10 (14.3%)	0.741
Cardiac events	3 (2.8%)	3 (5.7%)	0	
Cerebrovascular event	5 (4.6%)	0	5 (8.9%)	
CCF	7 (6.4%)	3 (5.7%)	4 (7.1%)	
PVD	2 (1.8%)	1 (1.9%)	1 (1.8%)	
RRT	11 (8.7%)	10 (17.5%)	1 (1.4%)	**0.001**
All-cause mortality	31 (24.4%)	12 (21.1%)	19 (27.7%)	0.427
Annual rate of decline of eGFR (Delta eGFR) (ml/min/1.73m^2^/year)	– 1.1 (– 4.2 to 0.33)	– 1.37 (– 4.2 to -0.034)	– 0.93 (– 4.2 to 1.2)	0.428
Follow-up, months	42 (23–66)	49 (30.5–81.5)	36 (22–54.6)	**0.013**

Events are expressed as numbers (percentage). *p*-value by Chi-Square test

Follow-up is expressed as median (interquartile range). *p*-value by Mann–Whitney *U* test

NFCVE-Non-fatal cardiovascular events- composite of cardiac events, cerebrovascular events, CCF, and PVD

Cardiac event- composite of non-fatal myocardial infarctions, acute coronary syndrome, coronary revascularizations, and non-fatal cardiac arrest. CCF-new diagnosis of congestive cardiac failure (CCF) and hospital admissions with CCF exacerbation. PVD-new diagnosis of peripheral vascular disease

The bold values indicate a statistically significant *p*-value i.e. *p*-value of 0.05 or lower

*RRT* commencing renal replacement therapy

When looking specifically at the indication for patients to undergo intervention in the revascularization group there were no major differences in demographics (Table 4). Annual mortality and RRT end-points in the 4 sub-groups were 4.6% and 6.1% for those revascularized primarily for hypertension, 5.7% and 4% for those requiring revascularization for deteriorating kidney function, 14.7% and 4.4% for the small number [6] of patients revascularized for acute heart failure syndromes, whereas none of the 9 patients stented for incidental significant renal artery stenosis died or required RRT during an average follow up period of just above 4 years.

**Table 4:** Baseline characteristics and outcomes in the revascularized patients based on indications for revascularization

Variable	All revascularized patients (*n* = 57)	Hypertension (*n* = 17)	Loss of renal function (*n* = 25)	Heart failure syndrome (*n* = 6)	High grade lesion (*n* = 9)	*p*-value
Age, years	68 (61–76)	64 (49–75)	70 (65–76)	71 (64–86)	68 (66–74)	0.396
Gender, female	35 (61.4%)	11 (64.7%)	14 (56%)	3 (50%)	7 (77.8%)	0.629
BMI, Kg/m^2^	27.1 (22.6–31.8)	27.4 (23.2–32.3)	27.2 (22.3–30.5)	24.8 (22.5–27)	27.3 (25.1–31.2)	0.824
Systolic BP, mm Hg	160 (139–181)	168 (154–193)	151 (136–172)	151 (107–171)	161 (135–179)	0.396
Diastolic BP, mm Hg	77 (66.5–99.5)	94 (76–105)	76 (60–91)	70 (66–80)	70 (68–87)	0.137
Smoking history	33 (57.9%)	8 (47.1%)	15 (60%)	4 (66.7%)	6 (66.7%)	0.720
Hypertension	57 (100%)	17 (100%)	25 (100%)	6 (100%)	9 (100%)	-
DM	13 (22.8%)	1 (5.9%)	8 (32%)	1 (16.7%)	3 (33.3%)	0.198
IHD	25 (43.9%)	7 (41.2%)	10 (40%)	4 (66.7%)	4 (44.4%)	0.689
CCF	21 (36.8%)	5 (29.4%)	7 (28%)	6 (100%)	3 (33.3%)	0.009
CVA	13 (22.8%)	3 (17.65)	7 (28%)	2 (33.3%)	1 (11.1%)	0.633
PVD	17 (29.8%)	4 (23.550	10 (40%)	1 (16.7%)	2 (22.2%)	0.512
Creatinine, micromol/L	127 (98–166)	122 (78–180)	123 (98–183)	130 (104–137)	138 (86–150)	0.906
eGFR, mL/min/1.73m^2^	40 (33.5–56)	46 (34.5–58)	40 (32.5–50)	41.5 (34–50)	39 (30–62)	0.824
UPCR, g/mol	36 (12.5–114)	22 (13–49)	53 (8.5–203)	51.2 (8–95)	22 (18–38)	0.864
*Outcomes*						
NFCVE	7 (12.3%)	2 (11.8%)	3 (12%)	1 (16.7%)	1 (11.1%)	0.989
Cardiac events	3 (5.7%)	2	1	0	0	
Cerebrovascular event	0	0	0	0	0	
CCF	3 (5.7%)	0	2	1	0	
PVD	1 (1.9%)	0	0	0	1	
RRT	10 (17.5%)	4 (23.5%)	5 (20%)	1 (16.7%)	0	0.486
All-cause mortality	12 (21.1%)	3 (17.6%)	7 (28%)	2 (33.3%)	0	0.285
Annual rate of decline of eGFR (Delta eGFR) (ml/min/1.73cm^2^/year)	– 1.37 ( – 4.2 to – 0.034)	– 1.37 ( – 1.89 to – 0.07)	– 1.06 ( – 4.5 to 0.92)	– 4.07 ( – 10.9 to – 0.15)	– 1.91 ( – 2.6 to – 1.1)	0.733
Follow-up (months)	49 (30.5–81.5)	46.5 (17.5–84)	59 (31–91)	27 (9.5–37)	49 (47–63.5)	0.227

Continuous variables are presented as median (interquartile range) and *p*-value of difference between groups by the Kruskal–Wallis Test. Categorical variables are presented as numbers (percentage) and *p*-values by Chi-square test

*BMI* body mass index, *BP* blood pressure, *DM* diabetes mellitus, *IHD* ischaemic heart disease, *CCF* congestive cardiac failure, *CVA* cerebrovascular accident, *PVD* peripheral vascular disease, *uPCR* urine protein creatinine ratio

NFCVE-Non-fatal cardiovascular events- composite of cardiac events, cerebrovascular events, CCF, and PVD. Cardiac event- composite of non-fatal myocardial infarctions, acute coronary syndrome, coronary revascularizations, and non-fatal cardiac arrest. CCF-new diagnosis of congestive cardiac failure (CCF) and hospital admissions with CCF exacerbation. PVD-new diagnosis of peripheral vascular disease. *RRT* commencing renal replacement therapy

Table 4 shows changes in blood pressure control in the patients who underwent revascularization. In the overall group of 57 patients there was an improvement in blood pressure from 160/77 mmHg pre-revascularization to 137/74 mmHg one year post intervention with no change in the number of anti-hypertensive medications required. Blood pressure fell in all 4 sub-groups, and among the 17 patients specifically revascularized for hypertension there was an early drop in blood pressure one month post procedure from 168/94 mmHg to 144/82 mmHg with a decrease in the mean number of agents required by 12 months from 5 to 3, with a significant improvement in diastolic blood pressure but minimally improved systolic blood pressure (163/75) at this timepoint (Table 5).

**Table 5:** Changes in blood pressure and blood pressure agents across groups over follow up

Variable	All revascularized patients (*n* = 57)	Hypertension (*n* = 17)	Loss of renal function (*n* = 25)	Heart failure syndrome (*n* = 6)	High-grade lesion (*n* = 9)
Systolic BP, mm Hg (57) Pre-Vasc	160 (139–181)	168 (154–193)	151 (136–172)	151 (107–171)	161 (135–179)
Diastolic BP, mm Hg Pre-Vasc	77 (66.5–99.5)	94 (76–105)	76 (60–91)	70 (66–80)	70 (68–87)
Number of antihypertension agents Pre- Vasc	3	4	3	3	3
Systolic BP, mm Hg (53) Immediate Post-Vasc	145 (131–155)	144 (131–157)	145 (131–151)	135 (109–141)	151 (144–157)
Diastolic BP, mm Hg Immediate Post-Vasc	74 (66–83)	82 (70–87)	74 (66–80)	71 (57–80)	71 (66–75)
Number of antihypertension agents Immediate Post-Vasc	3	3	3	3	3
Systolic BP, mm Hg (43) Post-Vasc 1 year	137 (152–166)	163 (136–183)	148 (137–159)	140 (122–152)	162 (148–180)
Diastolic BP, mm Hg Post-Vasc 1 year	74 (68–85)	75 (62–82)	75 (68–90)	70 (65–74)	76 (70–88)
Number of antihypertension agents Post-Vasc 1 year	3	3	3	4	3
Systolic BP, mm Hg (37) Post-Vasc 2 year	140 (128–154)	153 (141–160)	140 (122–150)	117 (112–125)	140 (130–150)
Diastolic BP, mm Hg Post-Vasc 2 year	74 (60–85)	85 (76–97)	77 (62–84)	74 (66–74)	60 (58–70)
Number of antihypertension agents Post-Vasc 2 year	3	3	3	4	3
Systolic BP, mm Hg (20) Post-Vasc 3 year	135 (124–153)	146 (141–154)	133 (124–135)	143 (97–189)	130 (128–135)
Diastolic BP, mm Hg Post-Vasc 3 year	79 (69–93)	87 (79–98)	72 (65–86)	79 (59–99)	70 (70–75)
Number of antihypertension agents Post-Vasc 3 year	3	3	2	–	3

*BP* blood pressure

The 25 patients revascularized primarily because of deteriorating renal function had an average pre-procedure rate of eGFR decline of -5.0 ml/min/1.73m^2^/year. This improved in 18 (72%) patients and the annual rate of eGFR decline improved to  – 1.3 ml/min/1.73m^2^/year (Table 6), with 20% of this group requiring RRT.

**Table 6:** Outcomes according to clinical presentation

Renovascular hypertension			
Variable	Pre-revascularization (*n* = 17)	One month post revascularization (*n* = 15)	One year post revascularization (*n* = 12)
Systolic BP, mm Hg	168 (154–193)	144 (131–157)	163 (136–183)
Diastolic BP, mm Hg	94 (76–105)	82 (70–87)	75 (63–82)
Number of agents	5 (4–5)	3 (2–3)	3 (2–4)

Ischaemic nephropathy			
Variable	Pre-revascularization (*n* = 25)	Post revascularization (*n* = 25)	*p*-value
*Delta eGFR ml/min/1.73m*^*2*^*/year*			
Median (IQR)	– 5.01 ( – 9.2 to – 3.2)	– 1.28 ( – 3.2 to 0.56)	0.004
Follow-up duration (years)			
Median (IQR)	3.2 (2.4–4.9)	2.7 (0.78–4.7)	0.172

Fifty percent of the 6 patients revascularized for a heart failure syndrome either died or required RRT during follow up, whereas the 9 patients revascularized because they were considered to have high-risk renal artery stenosis lesions, but no clinical syndrome, fared well with a significant fall in blood pressure at 3 years (161/70 falling to 130/70) and no mortality or requirement for RRT during follow up.

## Discussion

In this study of our centre’s practice, the decision of the multidisciplinary team led to the revascularization of 45% of patients with a diagnosis of renal artery stenosis in those with significant renal artery stenosis lesions. Since the neutral results of the ASTRAL and CORAL trials, our centre has recognised the importance of considering the mode of clinical presentation of atherosclerotic renovascular disease patients, and the fact that more careful case selection can result in positive outcomes for a minority of patients. Amongst those revascularized the main indications were deteriorating renal function, severe hypertension, a heart failure syndrome or the presence of a high-risk lesion.

Our study suggests that when considering the outcomes of patients with atherosclerotic renovascular disease it is important to focus these in relation to the clinical presentation of the patient. For example, although patients with renal artery stenosis and acute heart failure may also have declining kidney function and severe hypertension, important positive outcomes would be both survival and reduction in number of heart failure hospitalisations. This point was illustrated by the positive renal functional outcome of patients who were revascularized primarily because of deteriorating renal function. Overall, 72% showed an improvement in the slope of eGFR decline, with this reducing from  – 5.0 pre-procedure to  – 1.3 ml/min/1.73m^2^/year, although 20% of these patients eventually required RRT. A similar study from our centre which predated the establishment of the multidisciplinary team found revascularization was associated with reduced risk of progression to ESKD (HR = 0.35) in those with rapid decline in eGFR and bilateral renal artery stenosis ≥ 70% [17]. A similar study from Denmark evaluated the effect of revascularization in those with severe atherosclerotic disease and high-risk clinical presentation. In the subgroup of 63 patients with rapidly deteriorating renal function at baseline, revascularization was associated with an overall improvement in eGFR of 7.8 mL/min per 1.73m^2^ (95% CI, 4.5–11.1; *P* < 0.001) at 3 months, indicating some reversibility of renal dysfunction. At last follow up (median of 23.9 months), the eGFR was unchanged or had improved compared to baseline in 85% of those successfully treated in this subgroup [18].

In our study, patients revascularized for a primary indication of hypertension did have an immediate improvement in blood pressure control and a reduction in the number of anti-hypertensive agents at one year. Indeed, an immediate drop in both systolic and diastolic blood pressure was seen in the majority of revascularized patients. This is important as a recent meta-analysis of participants with or without prevalent cardiovascular disease (stroke, myocardial infarction or ischaemic heart disease) has shown that even a modest reduction of 5 mmHg in systolic blood pressure reduced the risk of major cardiovascular events by 10%, irrespective of a previous diagnosis of cardiovascular disease [19]. Due to the associated comorbidities our atherosclerotic renovascular disease patients were high risk for cardiovascular events. Our findings are consistent with a meta-analysis reporting that balloon angioplasty of renal artery stenosis results in a small improvement in diastolic blood pressure and a decrease in the mean number of anti-hypertensive agents required [20].

Other observational studies have shown that renal revascularization in those with atherosclerotic renovascular disease  and CKD can improve or stabilise renal function and preserve renal tissue. A study by Watson et al. illustrated that renal artery stenting in those with CKD and obstructive atherosclerotic renal artery stenosis resulted in preservation of renal size on ultrasound with a length of 10.4 ± 1.4 cm at baseline and 10.4 ± 1.1 cm after a mean follow up of 20 months [21].

Some observational studies have shown cardiovascular benefit from revascularization with reduced stroke and decompensated heart failure. A study by Ritchie et al. [6] from our centre included 467 patients with atherosclerotic renal artery stenosis and found that revascularization was associated with reduced risk for death ( HR, 0.4; 95% CI, 0.2–0.9; *P* = 0.01) and cardiovascular events (HR, 0.23; 95% CI, 01–0.6; *p* = 0.02) in patients with high-risk clinical presentations. There was also a reduction in the number of admissions for heart failure. For patients with previous acute pulmonary oedema the hazard ratio for future hospital admission in revascularized patients compared to those treated medically was 0.51 (0.08–3.30, *p* = 0.48).

A similar study from the Mayo Clinic compared the impact of either medical treatment or renal revascularization in 100 patients with renal artery sclerosis and coexistent heart failure on clinical outcomes. The groups were equally split 50/50. Stenting was associated with significant decrease in the New York Heart Association Functional Class (1.9 ± 0.8 versus 2.6 ± 1.0, *P* < 0.04) and a fivefold reduction in the number of subsequent heart failure admissions [22]. A limitation of many of these studies is the relatively short follow-up period averaging 3-5 years. Dregoesc et al. followed up 65 patients with atherosclerotic renovascular disease who underwent revascularization for a median of 10 years [23]. The study only included individuals with a renal artery stenosis lesion above 70% with some degree of CKD and/or uncontrolled hypertension. One year post stenting fewer patients had CKD as compared to baseline (35.3% vs. 56.9%, *P* = 0.01), and blood pressure also improved with 81.5% having controlled blood pressure compared to 12.3% at baseline. Mortality rates were higher in those who failed to improve from a blood pressure or CKD perspective. Post revascularization CKD class 3b–5 (OR 5.8; 95% CI 1.5–27.9; *P* = 0.01), and post revascularization uncontrolled hypertension (OR 8.9; 95% CI 1.7–63.5; *P* = 0.01) were associated with long-term mortality independent of diabetes mellitus and coronary artery disease. This would suggest that improvement in blood pressure control and renal function may have long term impact on the survival of patients with atherosclerotic renovascular disease. It also emphasises the importance of appropriate screening, diagnosis, and timely intervention in high-risk atherosclerotic renovascular disease patients rather than a blanket approach as previously seen in large RCTs which did not take into consideration the clinical presentation in these patients.

As mentioned earlier, the group in Lyon had a similar approach to our renovascular multidisciplinary team and demonstrated a significant improvement in blood pressure control in revascularized patients with no severe adverse events [7]. The therapeutic decision of the Lyon team was based on those with renal artery stenosis with a peak systolic velocity > 180 cm/s, and outcomes were considered in accordance with the 2013 AHA along with the 2017 ESC guidelines. The majority of those revascularized (78%) would have been recommended to have this treatment by the AHA guidelines but very few by the ESC guidelines. We also considered the guidelines, but each patient was discussed on a case by case basis and a patient-centred approach was taken in relation to the potential benefit of revascularization, particularly focusing on clinical presentation. Nonetheless, our criteria favouring revascularization are consistent with international guidelines including deteriorating renal function, poorly controlled hypertension despite multiple agents, heart failure syndromes and very high-risk asymptomatic lesions.

Many of our patients who did not undergo revascularization had > 50% renal artery stenosis but did not have any features of declining kidney function, uncontrollable hypertension or a heart failure syndrome; others were either of advanced age or the kidney beyond the stenosis was atrophic. However, 9 patients were revascularized because they had critical anatomical renal artery stenosis but without a high-risk clinical syndrome, and 7 of these patients benefitted with an improvement in blood pressure control, illustrating that selection of cases with either critical bilateral renal artery stenosis or very severe renal artery stenosis in a solitary kidney can still be worthwhile. Given that all 6 patients revascularized for heart failure had no further admissions, we could conclude that 45/57 (79%) of patients referred for revascularization after multidisciplinary team review did actually benefit from the procedure in some way.

When renal artery stenting is performed by experienced professionals the complication rate approaches 2% with the most common complications related to access, similar to our findings. Other complications such as cholesterol embolization, renal artery rupture/dissection, retroperitoneal haematoma, contrast-related injury, renal infarct and death are well recognised [24, 25].

In the ASTRAL trial the rate of major adverse events was 9% with 2 deaths [4]. In the CORAL trial [5] the renal artery damage rate was 4% similar to our findings of 3.5%. Our cohort however had a higher rate of cholesterol embolization (5.2%) than the 1.2% reported in CORAL, but this may be due to the clinically based diagnoses rather than the angiographically proven ones as seen in CORAL. However, in all of our cases there were no long-term sequelae from the embolization.

The important role of the multidisciplinary team in decision making has been illustrated in other specialities such as oncology [26] and immunology [27] in dealing with complex medical conditions with a lack of clear evidence. It also plays a role in considering the risk of complications in complex interventions such as spinal surgery [28].

Magnetic resonance angiography was the commonest screening modality amongst our cohort (57%), closely followed by computed tomography angiography. In other studies, doppler ultrasound has been used as the first line screening method in view of its sensitivity, specificity and relatively low cost [29]. However it requires dedicated in-centre expertise which is currently lacking in the UK.

There are several limitations to our study including its single-centre and retrospective nature, and because of the latter, selection bias and confounding by indication cannot be excluded. Despite the five-year duration of our study, patient numbers were relatively small. The final treatment decision was by consensus and patient-centred and not randomised or strictly following established guidelines. However, our decision-making process for revascularization was in keeping with the expert consensus view published after the Kidney Disease Improving Global Outcomes (KDIGO) meeting in 2020 [13] recommendations largely endorsed by a further expert group in a recent scientific statement on behalf of the American Heart Association [30]. Another limitation is that there was no control group of patients, for whom a multidisciplinary team meeting was not undertaken, but the study does suggest the use of a multidisciplinary team in identifying patients who will benefit from intervention. Ideally, a propensity-matching analysis might have strengthened the findings but the small cohort size did not allow for this.

**Fig. 2:**
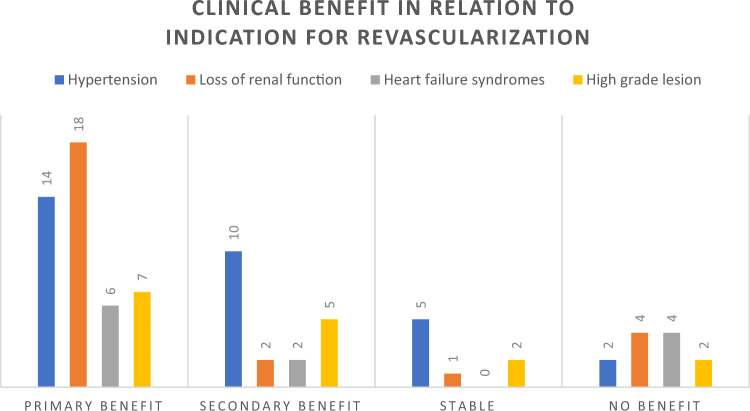
Clinical benefit in relation to indication for revascularization

In conclusion, with careful selection of patients based on a consensus renovascular disease multidisciplinary team decision that considered not only the physiological significance of the renal  artery stenosis lesion and the size of the kidney beyond the renal artery stenosis lesion, but also whether the patient had a high-risk clinical presentation, we have been able to illustrate that the majority (79%) of patients so treated had a clinical benefit. However, the nature of this clinical benefit was unsurprisingly linked to the nature of the clinical presentation of the patient (Fig. 2).

## Data Availability

The data underlying this article will be shared on reasonable request to the corresponding author.
