# Correction to: Improving outcomes in atherosclerotic renovascular disease: importance of clinical presentation and multi‑disciplinary review

**DOI:** 10.1007/s40620-024-02044-0

**Published:** 2024-08-05

**Authors:** Áine M. de Bhailis, Edward Lake, Constantina Chrysochou, Darren Green, Rajkumar Chinnadurai, Philip A. Kalra

**Affiliations:** 1 Department of Renal Medicine, Salford Royal NHS Foundation Trust, Salford, M6 8HD UK; 2 Faculty of Biology, Medicine and Health, University of Manchester, Manchester, UK; 3 Department of Vascular Interventional Radiology, Manchester University NHS Foundation Trust, Manchester, UK

**Correction to: Journal of Nephrology (2024) 37:1093–1105** 10.1007/s40620-024-01902-1

In the original article was reference 22 but duplicate of 6 so now 6.

In original article was reference 25 but a duplicate of 7 so now 7.

In the reference section of the original article, Had Salford Royal inappropriately inserted in last version.

The original article has been corrected.

